# Successful Treatment of a Rare Complication of Varicella Pneumonitis in an Immunocompromised 11-Year-Old Child Using Pooled Intravenous Immunoglobulin

**DOI:** 10.7759/cureus.71582

**Published:** 2024-10-16

**Authors:** Hadi A Helali, Ahsan A Saeed, Alaa Waleed, Jaidev R Nath, Moza Alhammadi

**Affiliations:** 1 Department of Pediatric Neurology, Al Jalila Children's Specialty Hospital, Dubai, ARE; 2 Department of Medicine, Mohammed Bin Rashid University of Medicine and Health Sciences, Dubai, ARE; 3 Department of Pediatrics, Al Jalila Children's Specialty Hospital, Dubai, ARE; 4 Department of Infectious Diseases, Al Jalila Children's Specialty Hospital, Dubai, ARE

**Keywords:** acyclovir therapy, immunocompromised patient, intravenous immunoglobulin (ivig), methotrexate-induced complications, varicella pneumonia

## Abstract

Varicella-zoster virus, or herpes zoster virus, is a human alphaherpesvirus. It causes varicella (chicken pox) and herpes zoster (shingles). Though a relatively common and benign childhood illness, it can lead to severe disseminated infections in immunocompromised patients. Varicella pneumonitis is a common complication of varicella found in adults but is rare in children. As such, we present a rare case of an 11-year-old immunosuppressed male on methotrexate who developed a disseminated varicella infection resulting in pneumonia. Methotrexate is not known to cause immunosuppression, as compared to infliximab, which is similarly indicated for rheumatoid autoimmune conditions. He was treated with high-dose acyclovir and pooled intravenous immunoglobulin (IVIG). Due to the efficacy of high-dose acyclovir in immunocompromised patients with varicella-zoster and modern preparations of IVIG containing high titers of zoster immunoglobulins, the treatment allowed the patient to recover fully.

## Introduction

Varicella-zoster virus (VZV) is a human alphaherpesvirus that causes varicella (chicken pox) and herpes zoster (shingles). Varicella is a common childhood illness, typically presenting with fever, viremia, and vesicular lesions scattered across the skin [1]. The epidemiology of VZV reveals a worldwide geographical distribution, with a greater prevalence in temperate regions (it is prevalent in almost all children under 10) and a lower prevalence in tropical regions (approximately less than 50% of children infected) [2]. Typically, reinfection in children is uncommon, but immunocompromised children reportedly have recurrent episodes of disseminated varicella [2,3].

VZV infection in children is usually benign and self-limited, but the course of varicella infection in the immunocompromised population is usually much more severe. In such patients, there is rapid dissemination into the organs, such as the lungs and the central nervous systems [3]. Immunocompromised individuals are also more likely to require ICU admission and mechanical ventilation and have a higher risk of mortality [4]. Pneumonia is a common morbidity of varicella in adults but is a rare complication in children, with 4.3 pneumonia complications per 10,000 cases of varicella infections [5].

Conventional guidelines establish VZV intravenous immunoglobulins (VariZIG) as standard treatment, but alternatively, pooled intravenous immunoglobulins (IVIGs) can be used, which contain a mix of immunoglobulins including varying titers of varicella-zoster immune globulin [6,7]. Immunoglobulin therapy is typically coupled with antiviral therapy, such as acyclovir, famciclovir, and valaciclovir [4]. Herein, we describe an 11-year-old Immunocompromised male with juvenile idiopathic arthritis (JIA) who developed severe disseminated varicella, which was successfully treated with pooled IVIG and high-dose acyclovir.

## Case presentation

The patient is an immunocompromised 11-year-old child with JIA. The patient had not yet received his childhood vaccine regimen for varicella (the VZV vaccine is given at age 12 in his home country). He was on weekly methotrexate (15 mg orally) for his JIA resulting in immunosuppression.

**Figure 1:**
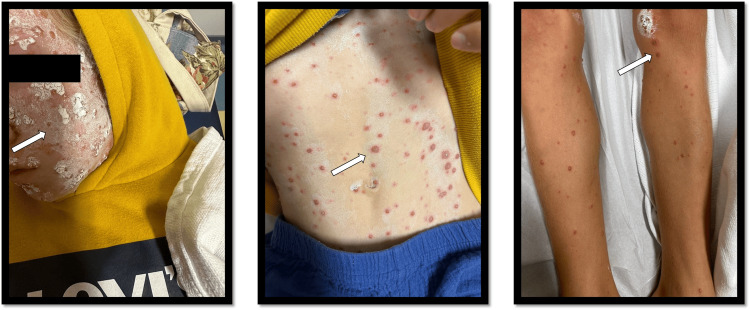
Child's initial clinical presentation in the emergency department (white arrows indicate vesicles on the body)

The patient presented to the emergency department with a history of fever and rashes that started five days before admission (Figure 1) and was admitted as a case of varicella. The patient was started on acyclovir (10 mg/kg/day), and methotrexate was withheld; VariZIG was not given as per the hospital guidelines stating not to administer it if the patient presented 72 hours after the onset of symptoms. Cultures (from blood and urine) were negative except for the varicella antigen polymerase chain reaction (from skin lesion biopsy), which was positive.

**Figure 2:**
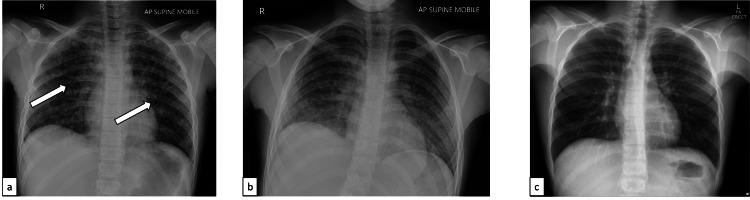
Patient's chest X-rays showing multiple small ill-defined nodular opacities noted in both lung fields. (a) Before IVIG (white arrows). (b) After IVIG. (c) After three months during follow-up IVIG: intravenous immunoglobulin

On the third day of admission, upon progressively worsening cough (but did not require oxygen supplementation or intubation and mechanical ventilation), a chest X-ray was done, which showed bilateral multiple small alveolar opacities in the lungs (Figure 2a). Afterward, labs were repeated, showing an elevated C-reactive protein and an increased elevation of liver enzymes (aspartate transaminase and alanine aminotransferase) (Table 1). Simultaneously, the child had new eye lesions (seen by a naked eye exam and fundoscopic exam by an ophthalmologist). The acyclovir dose was increased from 10 to 20 mg/kg/day. Topical acyclovir eye drops were added, and the patient received one dose of pooled IVIG at 1 g/kg.

**Table 1:** Laboratory result trend throughout patient’s treatment journey SGOT: serum glutamic oxaloacetic transaminase; AST: aspartate aminotransferase; mg: milligram; L: liter; U: unit; SGPT: serum glutamic-pyruvic transaminase; ALT: alanine transaminase; ED: emergency department; IVIG: intravenous immunoglobulin

Time when laboratory tests were done	C-reactive protein (normal range: 0-5 mg/L)	SGOT AST (normal range: 0-33 U/L)	SGPT ALT (normal range: 0-26 U/L)
Two months before the acute presentation: done in the clinic	<1.0	26.0	12.0
On the day of the acute presentation in the ED	33.4	73.0	68.0
On the first day of inpatient admission, after the administration of the low dose of acyclovir	73.9	58.0	48.0
On the third day of inpatient admission	79.7	116.0	82.0
On the seventh day of inpatient admission, after treatment with pooled IVIG and the high dose of acyclovir	5.1	50.0	94.0

Following the changes to the treatment, clinical improvements were noted, with shrinking of the alveolar opacities (Figure 2b) and normalization of the previous abnormal labs. The child was discharged on oral acyclovir (616 mg/day for 10 days). Follow-up after three months showed complete resolution of the rashes on the skin and complete resolution of the previously seen abnormalities on repeated chest X-rays (Figure 2c).

## Discussion

JIA is an autoimmune condition for which the disease-modifying antirheumatic drug methotrexate is given. The mechanisms of methotrexate's immunosuppressive properties are relatively unknown, but an in vitro study by Genestier et al. suggests that methotrexate has apoptotic properties in activated T-cells, which may suggest immunosuppressive effects [8].

The literature shows that methotrexate does not typically clinically manifest immunosuppression, as a double-blind placebo-controlled trial by Giannini et al. of low-dosage methotrexate for resistant JIA showed only mild adverse effects. These adverse effects were mainly gastrointestinal and not immune-related [9]. In contrast, infliximab, also used for autoimmune rheumatoid diseases such as JIA, leads to impaired cell immunity due to circulating tumor necrosis factor-alpha inhibition. If administered during incubation of primary VZV, it can lead to a severe disseminated form of the disease [10].

In the patient reported in the current paper, the dissemination of varicella resulted from immunomodulation by the methotrexate coupled with the lack of vaccination for VZV, even though the patient was on immunosuppressive medication. A literature review by Papp et al. evaluating the safety and efficacy of vaccinations during immunosuppressive therapies suggests that for live attenuated vaccines, the vaccine be given before treatment if feasible or when treatment is stopped [11], which the patient in this report should have had but unfortunately did not.

Kakinuma and Itoh report a case of severe hemorrhagic varicella in an immunocompromised woman, where conventional intravenous acyclovir was ineffective. A decrease in clinical features was only seen upon administration of continuous acyclovir [12]. In another study by Balfour et al., a higher dosage of intravenous acyclovir, 1,500 mg/m^2^ of body surface area per day, was effectively used to halt the progression of varicella-zoster. Compared to the placebo, the treated group had a higher virus clearance rate in the disseminated cutaneous lesions [13].

As described in this case, due to a lack of VariZIG (and given that it is indicated to be given beyond 72 hours due to decreased efficacy), one dose of pooled immunoglobulin (IVIG) was given at 1 g/kg. Maranich and Rajnik found an increase in the mean concentration of zoster immunoglobulin from 3.07 to 3.83 in historical (prevaccination) and postvaccination batches of IVIG. They concluded that current IVIG formulations contain high levels of varicella-specific immunoglobulins. Thus, as an alternative, physicians can carefully substitute varicella-specific immunoglobulin preparations (i.e., VariZIG) with IVIG in high-risk patients [6].

A similar case of disseminated varicella treated with IVIG and acyclovir was reported in the literature. The patient was an eight-year-old immunocompromised boy undergoing chemotherapy for acute lymphoblastic leukemia. This case had a marked number of similarities to the presented case as this patient was also on methotrexate, had acute hepatitis, and was treated with IVIG and high-dose acyclovir. The authors of that case report concluded that a high dose of IVIG, early administration of acyclovir, and mechanical ventilation are adequate treatments [14].

## Conclusions

In conclusion, varicella pneumonitis is a rare complication of primary VZV infection in children, for which immunosuppression is a major risk factor. Immunomodulation caused by methotrexate in children for JIA is rarely reported in the literature compared with similar drugs such as infliximab. Pooled IVIG can be used as a safe and effective alternative in the absence of VariZIG due to modern-day lots having adequate varicella immunoglobulin titers. As seen by the clinical response of the presented case and the literature, the authors conclude that continuous/high-dose acyclovir, IVIG preparations, and, if needed, mechanical ventilation (in case of severe respiratory compromise) are effective treatment modalities for disseminated varicella infection (including varicella pneumonitis).
